# Experiences of Alzheimer’s disease and related dementia family caregivers on Reddit communities: A topic modeling and sentiment analysis

**DOI:** 10.36922/aih.3075

**Published:** 2024-07-30

**Authors:** Yulin Hswen, Jiangmei Xiong, Margaret Hurley, Thu T. Nguyen

**Affiliations:** 1 Department of Epidemiology and Biostatistics, University of California San Francisco, San Francisco, California, United States of America; 2 Bakar Computational Health Sciences Institute, University of California San Francisco, San Francisco, California, United States of America; 3 Department of Biostatistics, Vanderbilt University Medical Center, Nashville, Tennessee, United States of America; 4 John Snow Inc, Washington, D.C., United States of America; 5 Department of Epidemiology and Biostatistics, College Park, University of Maryland School of Public Health, District of Columbia, United States of America

**Keywords:** Alzheimer’s disease and related dementias, Alzheimer’s, Dementia, Caregiver, Reddit, Social media, Natural language processing, Sentiment analysis, Topic analysis, BERTopic

## Abstract

Alzheimer’s disease and related dementias (ADRD) are a spectrum of disorders characterized by cognitive decline, which pose significant challenges for both affected individuals and their caregivers. Previous literature has focused on patient family surveys which do not always capture the breadth of authentic experiences of the caregiver. Online social media platforms provide a space for individuals to share their experiences and obtain advice toward caring for those with ADRD. This study leverages Reddit, a platform frequented by caregivers seeking advice for caring for a family member with advice for ADRD. To identify the topics of discussion or advice that most caregivers seek and sought after, we employed structured topic modeling techniques such as BERTopic to analyze the content of these posts and use an intertopic distance map to discern the variation in themes across different Reddit categories. In addition, we analyze the sentiment of the Reddit postings using Valence Aware Dictionary and Sentiment Reasoner to deduce the degree of negative, positive, and neutral sentiment of the discussion posts. Our findings reveal that the topics that caregivers most frequently discuss and seek advice for were related to caregiver stories, community support, and concerns ADRD. Specifically, we aimed to reproduce an organic Reddit search of caregiving of abuse on family member, financial struggles, symptoms of hallucinations, and repetition in ADRD family members. These results underscore the importance of online communities for gaining a comprehensive understanding of the multifaceted experiences and challenges faced by ADRD caregivers.

## 1. Introduction

Alzheimer’s disease and related dementias (ADRD) are a group of neurodegenerative disorders that are characterized by impaired thinking and independence.^1^ In 2023, an estimated 6.7 million Americans aged 65 and older were living with ADRD.^2^ ADRD features a gradual development, and as the disease progresses, behavioral and cognitive functions decline, affecting memory, comprehension, language, attention, reasoning, and judgment.^3^ People living with ADRD experience a range of symptoms that interfere with daily life. Memory loss, confusion, and poor judgment are often the first signs that appear in the affected individuals.^4^ Overtime, as the disease progresses from mild to moderate and from moderate to severe, additional compounding symptoms arise such as withdrawal from social activities, shortened attention span, difficulty carrying out familiar tasks, impulsive behavior, changes in sleeping patterns, difficulty recognizing friends and family, and general physical decline.^4^ Intensive supervision and care become necessary for patients. Managing increasing symptoms and needs can be difficult and highly stressful for patients as well as their families and caregivers.

Depending on the patient’s circumstances and needs, caregiving settings available to ADRD patients vary widely, with options for in-home care, adult care centers, long-term care facilities, and hospice.^5^ Many patients transition between care settings, posing a challenge for families, caregivers, and providers to ensure coordinated, continuous care.^2^ In 2020, the total health-care costs for treatment of ADRD was estimated at $305 billion, most of which is attributed to the cost of care.^6^ However, indirect costs of care such as quality of living and informal caregiving are likely underestimated.^6^ Patients generally receive increasing care from family members and other unpaid caregivers as their disease progresses^2^. It has been estimated that 75% of caregiving given to a patient with Alzheimer’s disease is informal care provided by the patient’s family and friends^6^. In 2022, more than 11 million family members and unpaid caregivers provided an estimated 18 billion hours of care to people with ADRD.^2^ On top of the emotional burden of caring for a family member with declining cognitive ability, navigating the care system is time-consuming and costly, extending to family caregivers’ increased risk for negative mental and physical health outcomes.^2^

Family caregivers play a critical role in the quality of life of ADRD patients, yet their mental health and support circles have been understudied. Research on the experiences and needs of caregivers would not only benefit other caregivers in similar situations but also provide insight for health-care providers and researchers to understand and respond to the needs of these individuals.^7^ Existing studies have gathered data on caregivers’ well-being through methods such as questionnaires and interviews,^8,9^ and recent trends saw the emerging data analysis of caregiver experiences from various social media platforms – an investigation strategy that could avoid response bias.^10,11^ Social media platforms allow for the collection of large amounts of content in the form of users’ health-care knowledge, experiences, symptoms, products, doctors, and medicines for research purposes^11^ and offer insights from otherwise hard-to-reach populations.^12^

For instance, empirical research is increasingly turning to Reddit as a source of information due to its large user base and highly specialized subreddit communities.^12^ Compared to other social media platforms, Reddit has a lower noise-to-information ratio, which makes it ideal for people who are caring for family members with ADRD. Recent studies have begun to analyze Reddit posts to research the experiences and challenges of caregivers of patients with Alzheimer’s disease and other forms of dementia.^7,13^ This paper builds on existing studies and focuses on the important topics of caregivers of family members with Alzheimer’s disease. Data are analyzed with topic modeling and sentiment analysis. The analysis reveals the major concerns of Alzheimer’s patient’s family caregivers, offering insight into future care and assistance to this population.

## 2. Data and methods

### 2.1. Data source and cleaning

To mimic an organic search on Reddit, “caregiving for a family member with dementia” was entered into the search box and searched on Reddit. Using the Reddit API, the most relevant posts were extracted. Reddit API allows for the return of information within a post, including user ID, post title, post content, post time, and all comments. In total, 1151 replies were collected.

Text from posts and comments were preprocessed before topic analysis. The goal of preprocessing is to keep words that carry essential meaning. Thus, we remove stopwords using NLTK stopwords dictionary.^14^ Stopwords such as “a” and “the” are words that do not convey important information and add little to the comprehension of the text.^15^ This step denoises the text input. Next, all words were lemmatized using SpaCy.^16^ In this step, all words were swapped with their lemma, so that only the content of the words remained. This step ensures that words in different forms are counted as one in the topic analysis.

### 2.2. Topic modeling

To understand the top topics within the Reddit comments, we performed topic modeling, a machine learning-based classification method for texts.^17^ In this study, we used BERTopic, a sentence-transformers model, for extracting embedded document. Compared to previous methods such as Latent Dirichlet Allocation (LDA) modeling,^18^ BERTopic incorporates the semantic context of words and further fine-grained the method by considering the varying word semantic distance distributions.^19^ Similar to the user interface of other topic models, it outputs topic assignment for each comment, as well as the top words of each topic. The top words help us interpret the topics of the comments, while topic assignment lets us see how popular each topic is, and it can also be used in the subsequent sentiment analysis.

Another difference between BERTopic and LDA modeling is that BERTopic determines the number of topics by the text, while LDA relies on a user-defined number of topics.^20,21^ Using BERTopic, we generated an intertopic distance map to determine the distance (difference) between the topics. An intertopic distance map represents each topic as a circle on Cartesian plane, whose coordinates represent semantic distance. If circles do not overlap, it is considered that the topics are well separated. If not, the topic model will be refitted with an adequately smaller topic number, and the intertopic distance map will be plotted again to see if the topics are well separated. The “step-size” of each refitting can vary depending on prior knowledge on the dataset. For example, in the case where no more than 20 topics are expected in the text, and BERTopic model identifies more than 100 topics, the “step size” can be 5 – 10 less topics for next refitting, until topic separation appears, or that number of topics is reduced to 20. After that, the “step size” can be 1 less topic for each refitting.

### 2.3. Sentiment analysis

To understand the sentiment that a comment carries, we performed sentiment analysis, which quantifies positive and negative sentiment. We adopted the most widely used sentiment analysis, Valence Aware Dictionary for Sentiment Reasoning (VADER), for our purpose in this study.^20^ VADER is a rule-based model that summarizes lexical, grammatical, and syntactical features of text and quantifies the tone of sentiment into scores.^20^ Compound VADER scores are normalized from the raw VADER scores and span from −1 to 1, with a negative score representing negative sentiment, and vice versa. We followed the rule of thumb in VADER sentiment analysis and identified those with compound VADER scores <−0.05 as negative comments, −0.05 to 0.05 as neutral, and those with compound VADER scores >0.05 to be positive comments.

## 3. Results

### 3.1. BERTTopic modeling output

A total of 1151 comments were collected from 15 Reddit posts from our search results.^19^ Using BertTopic topic modeling and manual topic refinement, we categorized the comments into six topics and provide example comments for each topic in Table 1. Topic 0 was identified as “sharing caregiver stories,” topic 1 as “appreciation of online community,” topic 2 as “concerns of abuse of ADRD family member,” topic 3 as “financial struggles of caregivers,” topic 4 as “early symptoms of ADRD of family member,” and topic 5 as “symptoms of ADRD.” As seen in Table 1, the topic having the greatest proportion of discussions was topic 0 (*n* = 926), followed by topic 1 (*n* = 126), topic 2 (*n* = 33), topic 3 (*n* = 31), topic 4 (*n* = 22), and topic 5 (*n* = 13).

### 3.2. VADER (sentiment analysis) results

**Figure 1.**
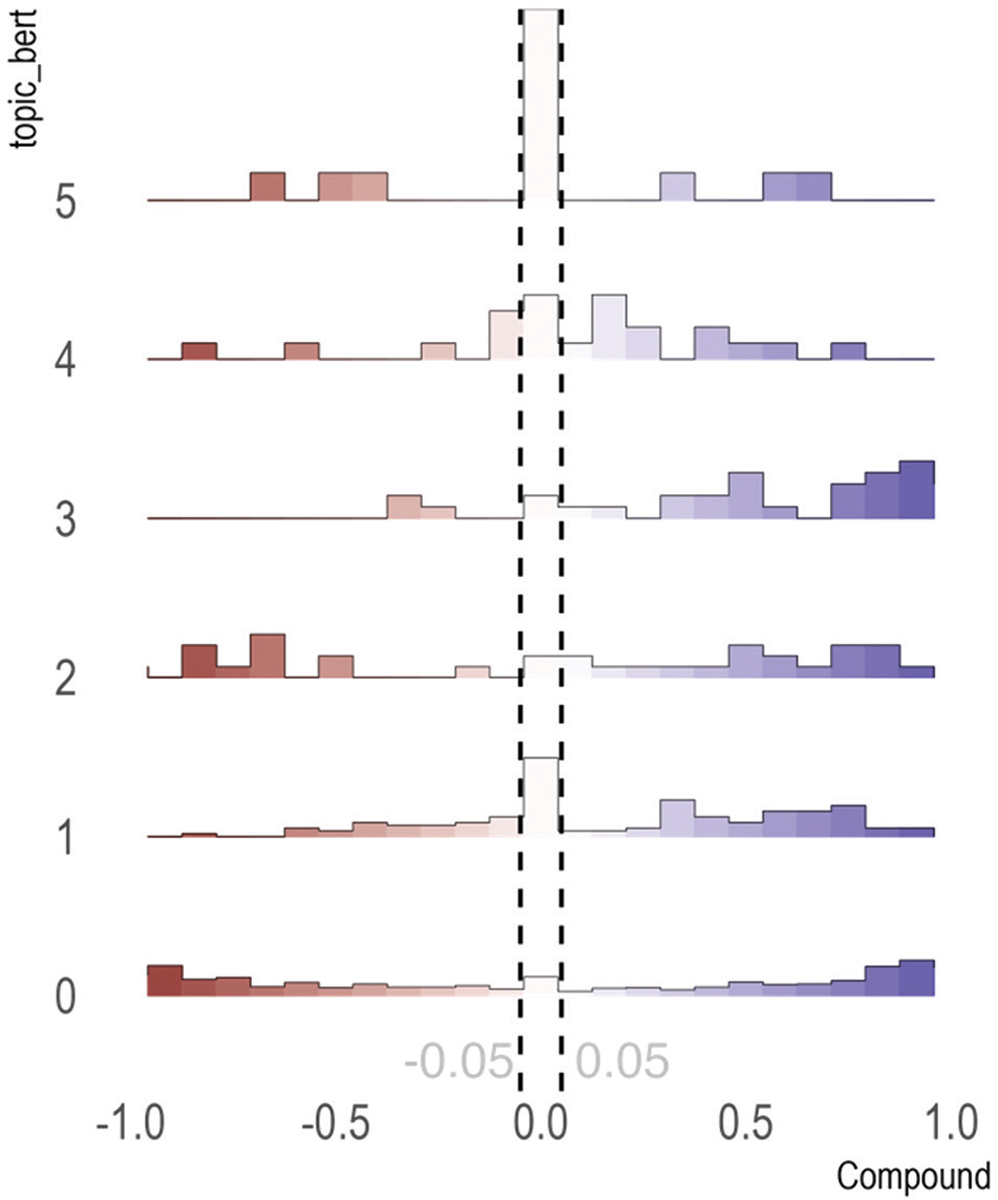
Sentiment distribution of topics. Source: By authors

**Figure 2.**
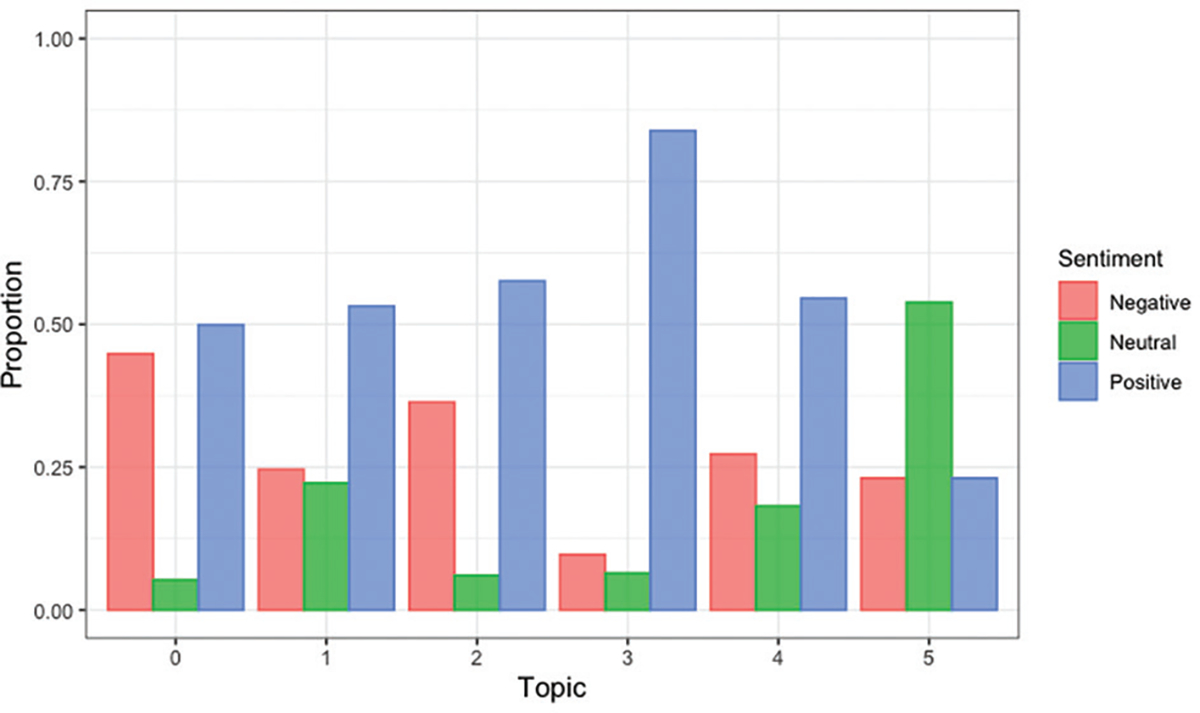
Proportion of negative, neutral, and positive sentiment across topics. Source: By authors

We used VADER to analyze the sentiment of the comments under each topic. Figure 1 describes the average VADER sentiment score of the retrieved posts’ texts for each topic. In Figure 1, the x-axis corresponds to the VADER compound score that ranges from −1 to 1, where x<−0.05 represents negative sentiment, −0.05<x<0.05 represents neutral sentiment, and x>0.05 represents positive sentiment. As described by the histogram bars in Figure 1, topic 3 is skewed to the right indicating more positive sentiment, while topics 1 and 3 are skewed to the left indicating more negative sentiment. Figure 2 provides a direct comparison of comment sentiment proportions. Topic 0 had relatively equal proportions of positive and negative sentiment, whereas topic 5 had the most proportion of neutral sentiment and topic 3 had the highest proportion of positive posts.

The top words in each topic are displayed in Table 2. Topic 0 was the largest topic of posts and manually labeled as “shared stories by caregivers.” This topic included stories that ADRD caregivers shared with other ADRD caregiving users on Reddit. Comments included personalized experiences of their family member having ADRD symptoms, describing in detail specific cases. Top keywords included specific family members, such as “mom” and “dad.” As shown in Table 1 and Figure 1, 44.8% of the posts were negative and 53.2% of posts had a positive sentiment.

Topic 1 was manually labeled as “appreciation of online community.” This topic included comments in which caregivers shared gratitude and thanks with other Reddit users, showcasing the benefit of these online communities. The top five keywords in Topic 1 were “thank,” “sorry,” “much,” “go,” and “share.” As shown in Table 1 and Figure 1, 24.6% of the posts had a negative sentiment and 53.2% had a positive sentiment.

Topic 2 was identified by BERTopic and manually labeled as “concerns of abuse for ADRD family members.” This topic included posts in which caregivers described fears of ADRD family members who were patient abuse at care facilities. The top five keywords in Topic 1 were “abuse,” “forgiveness,” “peace,” “abuser,” and “heart.” As shown in Table 1 and Figure 1, 36.4% of the posts in Topic 2 had a negative sentiment and 57.6% had a positive sentiment. Despite Topic 2 having a higher proportion of positive comments than negative comments, the negative posts had the highest mean negative VADER scores among all the topics (Figure 1).

Topic 3 was identified by BertTopic and manually labeled as “financial struggles of caregivers.” This topic included comments related to the financial situations and complexities faced in taking care of a family with ADRD by caregivers. The top five keywords in Topic 3 were “medicaid,” “state,” “care,” “asset,” and “qualify.” As shown in Table 1 and Figure 1, 9.7% of the comments in Topic 3 had a negative sentiment and 83.9% had a positive sentiment. Topic 3 had the highest proportion of positive comments as well as the highest mean positive VADER scores among all the topics (Figure 1).

Topic 4 was identified by BERTopic and manually labeled as “early signs of ADRD in family members.” This topic included posts in which caregivers discussed their family members’ early symptoms of ADRD. The top five keywords in Topic 4 were “hallucination,” “cat,” “see,” “real,” and “brain.” It appears that the first signs of ADRD in family members started to surface when they began to hallucinate and were neglecting their pet cats. As shown in Table 1 and Figure 1, 27.3% of the posts had a negative sentiment and 54.5% had a positive sentiment.

Topic 5 was identified by BERTopic and manually labeled as “symptoms of ADRD in family members.” This topic included posts in which caregivers discussed their family members’ most frequent symptoms of ADRD. The top five keywords in Topic 5 were “repeat,” “story,” “plan,” “get,” and “weekend.” As shown in Table 1 and Figure 1, 23.1% of the posts had a negative sentiment and 23.1% had a positive sentiment. For instance, family members with ADRD have symptoms of repeating stories and questions and forgetting plans for the weekend.

### 3.3. Intertopic distance map

**Figure 3.**
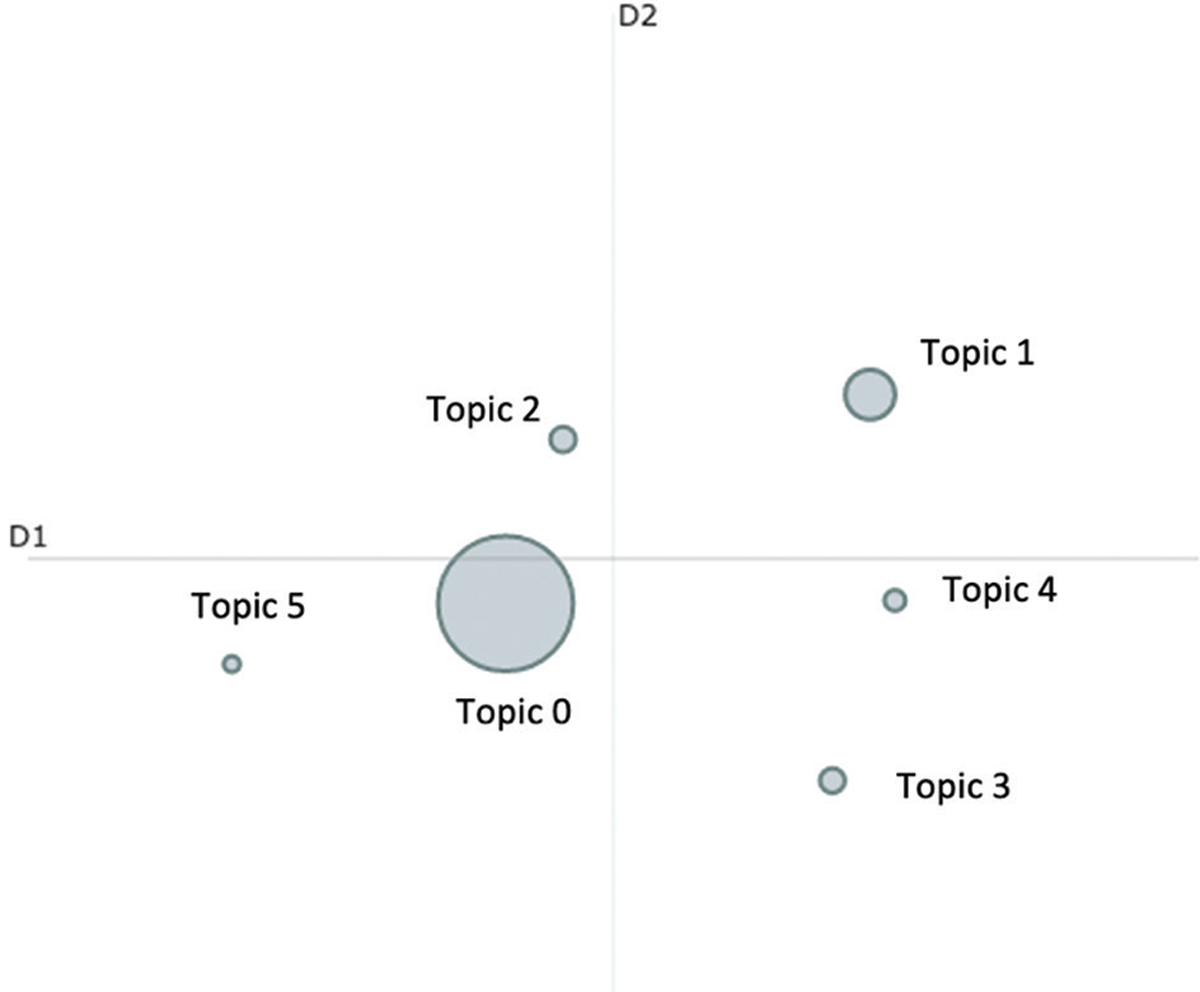
Intertopic distance map for all topics. Source: By authors

Figure 3 displays the intertopic distance map showing a visualization of the topics, with the area of the topic circles proportional to the number of words that belong to each topic and the distance between the topics representing the degree of difference between each topic. From Figure 3, we can see that all topics are well separated. Among the topics, smaller clusters can be observed, where topics 0, 2, and 5 are clustered, while topics 1, 3, and 4 are clustered to each other. The clustering and proximity of topics to one another indicate that the texts in these topic clusters were related to one other semantically. It also follows that topics that are further distanced share less similarity semantically.

## 4. Discussion

The objective of this study was to determine how Reddit provides online support to caregivers of ADRD. By performing an organic search and collecting the top Reddit postings, we revealed the most prominent topics of discussion among family caregivers of ADRD patients. These results bring to light to perspectives and experiences of caregivers and determine the sentiment of Reddit community forums. Overall, the sentiment of Reddit posts was neutral but leaned toward a positive skew because of the support that Reddit users who were family caregivers of ADRD patients received.

### 4.1. Building on previous research

Topic modeling has been frequently used to understand unstructured data such as those from social media. Previous systematic review has evaluated the use of topic modeling on social media and overall, it is effective at understanding themes but has its limitations.^21^ We employed standard tokenization and the removal of stop words, which is essential to the analysis of topics. We, however, did not evaluate discussions on social media that were multilingual. It is true that multilingual discussions exist on Reddit but, for the purposes of this study, we only collected texts that were in English. In addition, there are various topic models that can be used, which can dictate the interpretation of the data. However, based on this systematic review, LDA analysis is the most frequently used method as it provides the most robust and clean sectioning of data into topics.^21–24^ LDA topic analysis has also been used across various dimensions of health and countries.^23^ Furthermore, we employed a sentiment analysis tool VADER as an additional method in combination with topic analysis to further validate how caregivers feel toward caring a family member with dementia.^25–28^

### 4.2. Burden of ADRD on family members

Our findings point broadly to the substantial impact of ADRD on family caregivers. The physical, emotional, and mental stress of diagnosis extends beyond the patient and to those who care for them as the disease progresses. There is no cure for ADRD, so as patients’ cognitive abilities decline, family members become their advocates and care experts. The Reddit comments that we analyzed revealed high levels of distress among family members, which is associated with their increase in responsibilities, difficulty navigating care options, disheartening interactions with symptomatic loved ones, and the strain it puts on other areas of their lives. Some illustrative comments are given in the following:

*“I feel I have no life and my life revolves around taking care of my dad.”* (*Topic 0*)

*“I’ve worked as a nurse for many, many years, with the last 15 of those being in aged care specialising in care & it’s always hardest on the family.”* (*Topic 0*)

*“My Mom is the sole caregiver and it has been extremely hard on her as you’d expect. My Dad is in a long term care facility now and she’s there with him everyday. I feel like Alzheimers has taken away both of my parents, my Dad but also my Mom.”* (*Topic 1*)

*“I love my grandma so much and it’s always so disheartening to visit her in the state that she’s in.”* (*Topic 0*)

### 4.3. Reddit as a resource and form of support for caregivers

Despite the toll that the disease takes on caregivers, our findings revealed primarily positive comments in the Reddit forum. This points to the sense of community and support that the Reddit online community offers. For instance, when a user posts a comment on Reddit that shares about their caregiving experiences, challenges, or questions, the other users in the forum who have gone through similar caregiving situations respond by sharing their advice, empathy, and condolences. Several illustrative comments are presented below:

“*I’m so sorry you are so overwhelmed. I hope you have someone there you can talk to about this, and relatives to give you a break from caretaking.*” (*Topic 1*)

“*You have every right to feel overwhelmed to see her deteriorate in front of you and want the best care for her. It’s a total disruption to your life with added responsibilities to have to suddenly care for someone with*.” (*Topic 1*)

“Don’t be *too* hard on yourself if possible. And if you ever want to talk with (or at) someone that might be able to relate a bit you (and anyone else dealing with this kind of thing) are absolutely, seriously, earnestly, always welcome to message me.” (*Topic 1*)

“*Good luck. Please remember to forgive yourself for any frustration you feel. It is natural, normal, and would be weird for you not to have any*.” (*Topic 0*)

As family members manage numerous symptoms that progress over time and vary across ADRD patients, Reddit serves as a forum for caregivers to share tips and recommendations that they have learned. Navigating the care systems, symptoms, and treatment for ADRD is complex and daunting. For caregivers researching their loved ones’ symptoms and care situations, Reddit serves as a wealth of knowledge curated by those with first-hand experience. Our findings point to Reddit as a resource to medical for patient care management, supplementary to the treatment and advice that medical practitioners provide. Several illustrative comments are shown in the following:

“*My wife and I have been going through it with her dad for several years now. He has and is just now starting to have issues with urine control. We believe it’s a side effect from one of his meds. I advise you check her meds and make adjustments if you can.*” (*Topic 0*)

“*We are looking for a memory care facility rather than a nursing home and I recommend you research the same*.” (*Topic 0*)

“*After she started becoming more physical with me we decided to put her in a secured facility (so she wouldn’t wander off) It was great! They had activities and a routine for her which really eased my guilt for placing her there. Maybe that’s something you should consider*.” (*Topic 1*)

This study underlines the importance of Reddit as a resource for caregivers who may be looking for a forum for managing care, sharing experiences, and finding support.

### 4.4. Reddit as a resource for practitioners, researchers, and health-care organizations

While it is evident that Reddit forums act as a form of support and guidance for caregivers, our findings can also be applied in professional health-care settings. The sentiment analysis of recurring topics in Reddit threads related to Alzheimer’s disease informs health-care providers of some of the most common symptoms that caregivers struggle to manage. Reddit’s candid format brings unbiased perspectives and insight into the way that resources, tools, and educational services should be diverted in health-care settings for caregivers and patients. A representative feedback below illustrates this:

“*The hardest part for me has been dealing with the anger, paranoia, and confabulation that my mom presents with*.” (*Topic 1*)

## 5. Limitations

A limitation of this study is its reliance on Reddit for data, which, despite providing a rich dataset of caregiver experiences, might not capture the full spectrum of perspectives available on the array of social media platforms. Incorporating data from other platforms such as Facebook groups, Twitter, and other online forums could offer greater insight. Different platforms often cater to diverse demographics and feature varied caregiving experiences, which could enrich the overall analysis by presenting a broader range of insights. For this study, we focused on using Reddit data because of its specific forums for ADRD caregivers.

A second limitation of the current study is its cross-sectional design, which does not track changes in discussions and sentiments of ADRD caregivers over time. With the anonymous nature of Reddit, we are unable to track users longitudinally. A longitudinal study would provide valuable insights into how caregiving challenges and needs evolve, reflecting the dynamic nature of caregiver experiences as the disease progresses or in response to changes in social and health care that ADRD family members receive. Such an approach would allow for the identification of trends and shifts in caregiver concerns, offering a deeper understanding of the temporal dynamics at play. However, the anonymity of this social media data is also critical to capturing organic and honest personal caregiving experiences. Research should continue to prioritize privacy protections and confidentiality for this social media data.

## 6. Future directions

In future studies, new artificial intelligence (AI)-based approaches for the analysis of caregiver discussions should be explored. Specifically, transformer-based models, such as GPT-3 and its iterations, could provide a more nuanced understanding of context, sentiment, and emotional undertones. The next step in subsequent studies should be to test these new generative AI tools to determine whether they can match or surpass the performance of currently validated natural language processing techniques. This would enhance the field and also ensure that we evaluate what is the most effective AI tool in understanding unstructured human communications.

This study focused on the top posting on Reddit about caregiving for family members with ADRD and did not explore the variability of caregiving experiences and sentiments across different cultures, geographic locations, and demographic groups, including age, gender, and socio-economic status. The challenge with using Reddit is the preservation of users’ anonymity. On top of that, subanalyses specific to different demographics may illuminate the unique challenges and needs faced by caregivers from diverse backgrounds. Efforts to understand these differences are crucial for the development of targeted support and resources that are tailored to meet the specific needs of ADRD caregivers. Identifying distinct social factors among ADRD patient’s caregivers can help devise support that is inclusive and responsive to the diverse realities of caregivers worldwide.

Finally, comparing insights from social media data of ADRD caregivers with data from electronic health records and clinical notes concerning ADRD may provide further validation of these online data sources. This integrative approach would allow researchers to identify potential correlations between the topics and sentiments expressed in social media discussions and actual clinical outcomes. By mapping these discussions to specific stages of ADRD, it may be possible to discern patterns and trends that inform more effective caregiver support strategies. This method could prove invaluable in enhancing our understanding of how caregiver experiences and needs evolve in response to the progression of the disease and the efficacy of interventions.

## 7. Conclusion

This study uses sentiment and topic analysis, to disentangle posts on Reddit on how caregivers or patients themselves are self-managing care. Our findings can also be useful for bridging the gap between theoretical insights derived from social media discussions and actionable recommendations. Health-care providers can use this information to translate the nuanced understanding of caregiver experiences from these social media sources into improved support strategies and patient care interventions, ultimately benefiting both caregivers and patients by ensuring that the insights gained are effectively applied in health-care settings.

## Figures and Tables

**Table 1.** BERTopics, keywords, and sentiment from posts on caregiving for family members with ADRD on Reddit

Topic	Number of comments	Top 5 keywords	Topic interpreted	# Negative comments (%)	# Positive comments (%)	Example text
0	926	Get, go, time, thing, know	Sharing of caregiver stories	415 (44.8)	462 (49.9)	“*First, it was little things - but very noticeable - such as words. A few months later, it was people; she would confuse people from her past and the present. Then, she started hallucinating and making up stories about people that didn’t exist doing things with her she couldn’t have possibly done (like going to places that no longer existed in my hometown or visiting friends who were dead). Next, she completely forgot about my grandpa (who’d been dead for ten years at this point, and to whom she’d been married for over 50), and after that, she also forgot about her firstborn son, who had died 8 years before*.”
1	126	Thank, sorry, much, go, share	Appreciation of online community	31 (24.6)	67 (53.2)	“*I appreciate you sharing this. Lots of us are in the same boat with you*.”
2	33	Abuse, forgiveness, peace, abuser, heart	Concern of abuse on ADRD family member	12 (36.4)	19 (57.6)	“*Not if they don’t want to be. I feel sad for the person he is now because dementia really does change who you are, but I do not fault the family. They still see their abuser when they look at him since he still has the same face, and it doesn’t make sense to revictimize them so he can have extra company*.”
3	31	Medicaid, state, care, asset, qualify	Financial struggles of caregivers	3 (9.7)	26 (83.9)	“*How do you afford such care in America? Insurance only covers 28 days (classified as rehab) and the rest is out of pocket. And it’s very expensive. Soon the accounts are empty and they go on Medicaid, right? Eventually drying up all assets.*”
4	22	Hallucination, cat, see, real, brain	Early signs of ADRD in family member	6 (27.3)	12 (54.5)	“*The first thing we noticed was paranoia. It started as vague and even somewhat plausible and over time just got more and more extreme. She lives in assisted living now and pretty much everyone who visits her has said that they’re taking up dangerous extreme sports because that is no way for a person to die.*”
5	13	Repeat, story, plan, get, weekend	Symptoms of ADRD in family members	3 (23.1)	3 (23.1)	Two minutes later: “*Have you got plans for the weekend?*”

Abbreviation: ADRD: Alzheimer’s disease and related dementias.

**Table 2.** Top words by topic

Topic 0		Topic 1		Topic 2		Topic 3		Topic 4		Topic 5	
Count	926	Count	126	Count	33	Count	31	Count	22	Count	13

*Keyword*	*Frequency*	*Keyword*	*Frequency*	*Keyword*	*Frequency*	*Keyword*	*Frequency*	*Keyword*	*Frequency*	*Keyword*	*Frequency*

Get	0.038	Thank	0.425	Abuse	0.207	Medicaid	0.176	Hallucination	0.227	Repeat	0.289
Go	0.033	Sorry	0.190	Forgiveness	0.173	State	0.107	Cat	0.167	Story	0.286
Time	0.031	Much	0.122	Peace	0.139	Care	0.082	See	0.090	Plan	0.146
Thing	0.027	Go	0.095	Abuser	0.112	Asset	0.072	Hallucinate	0.075	Get	0.123
Know	0.025	Share	0.093	Heart	0.103	Qualify	0.069	Real	0.074	Weekend	0.119
Start	0.024	Lovely	0.090	Cut	0.095	Cost	0.063	Brain	0.071	Tell	0.118
Year	0.024	Say	0.082	Family	0.079	Pay	0.060	Look	0.055	Minute	0.115
Mom	0.023	Person	0.064	Deserve	0.079	Insurance	0.055	Even	0.052	Time	0.094
Say	0.022	Funny	0.063	Forgive	0.067	Social	0.053	Go	0.047	Question	0.085
Dad	0.021	Man	0.062	Tie	0.065	Cover	0.047	Dishwasher	0.047	Familiar	0.081

## Data Availability

Data will be available upon reasonable request.
